# Successful Bone Marrow Transplantation in a Patient with Acute Myeloid Leukemia Developed from Severe Congenital Neutropenia Using Modified Chemotherapy and Conditioning Regimen for Leukemia

**DOI:** 10.3390/hematolrep16010010

**Published:** 2024-02-26

**Authors:** Risa Matsumura, Shinji Mochizuki, Yusuke Morishita, Hiroko Hayakawa, Shuhei Karakawa, Hiroshi Kawaguchi, Satoshi Okada, Nobuyuki Hyakuna, Masao Kobayashi

**Affiliations:** 1 Department of Pediatrics, Hiroshima University Hospital, Hiroshima 734-8551, Japanmasak@hiroshima-u.ac.jp (M.K.); 2 Department of Pediatrics, Kochi University, Kochi 783-8505, Japan; 3 Department of Pediatrics, University of the Ryukyus Hospital, Okinawa 903-0215, Japan; 4 Japanese Red Cross Chugoku-Shikoku Block Blood Center, Hiroshima 730-0052, Japan

**Keywords:** severe congenital neutropenia, acute myeloid leukemia, hematopoietic stem cell transplantation

## Abstract

Severe congenital neutropenia (SCN) is characterized by chronic neutropenia with recurrent infections from early infancy and a predisposition to myelodysplastic syndrome/acute myeloid leukemia (AML). Allogeneic hematopoietic stem cell transplantation (HSCT) is the only curative treatment for patients with SCN who develop myelodysplastic syndrome/AML. We report an 8-year-old girl with SCN carrying an *ELANE* mutation that had been refractory to granulocyte colony-stimulating factor. The patient experienced recurrent infections and then developed AML. The counts of leukemic blasts that harbored both *CSF3R* and *RUNX1* mutations spontaneously decreased with antimicrobial therapy, leading to partial remission. After AML recurrence, HSCT was successfully performed using modified chemotherapy and a conditioning regimen. Serial donor lymphocyte infusions against mixed chimerism induced complete donor chimerism over 4 years without any infections or AML relapse. This case suggests the importance of carefully managing neutropenia-related infections, leukemia progression, and HSCT in patients with SCN developing AML.

## 1. Introduction

Severe congenital neutropenia (SCN) is a heterogeneous bone marrow failure syndrome characterized by chronic neutropenia with an absolute neutrophil count (ANC) of fewer than 500/µL and the arrest of myeloid maturation at the promyelocyte or myelocyte stage in bone marrow [1,2]. The most common genetic abnormalities in patients with SCN are heterozygous mutations in *ELANE*, which encodes neutrophil elastase [3]. Other genetic mutations reported in patients with SCN include *HAX1*, *G6PC3*, and *GFI1* mutations [4,5,6]. Patients with SCN are susceptible to infections, and they have an increased risk of developing myelodysplastic syndrome (MDS)/acute myeloid leukemia (AML) [7,8]. Allogeneic hematopoietic stem cell transplantation (HSCT) is the only curative treatment for patients with SCN who develop MDS/AML [9,10,11]. We present the case of an 8-year-old girl with SCN carrying an *ELANE* mutation who developed AML, in whom spontaneous partial remission (PR) was initially achieved following treatment of the infections. The patient successfully underwent bone marrow transplantation (BMT) with modified chemotherapy and a conditioning regimen for AML.

## 2. Case Report

A 4-day-old female newborn presented with skin infections and an ANC of 400/µL in peripheral blood. At 1 month old, she experienced recurrent skin infections with a low ANC. Bone marrow examination revealed arrested myeloid maturation at the promyelocyte/myelocyte stage. Then, the patient was treated with granulocyte colony-stimulating factor (G-CSF). The daily G-CSF dose was increased to 40 µg/kg when the patient was 2 months old; however, the ANC remained below 100/µL. G-CSF treatment was discontinued at 9 months of age because of refractoriness. The patient was diagnosed with SCN carrying a heterozygous gene mutation in exon 1 of *ELANE* [c.1A>G (M1V)] at 1 year of age. No family history of hematologic disorders or any other diseases associated with SCN or AML were identified. HSCT was proposed considering the risk of serious infections and/or progression to MDS/AML because of the refractoriness of G-CSF treatment; however, the patient’s parents refused HSCT because they were concerned about the risk of transplantation-related morbidity and mortality. Therefore, the patient experienced recurrent infections, including severe skin infections and liver abscess.

**Figure 1:**
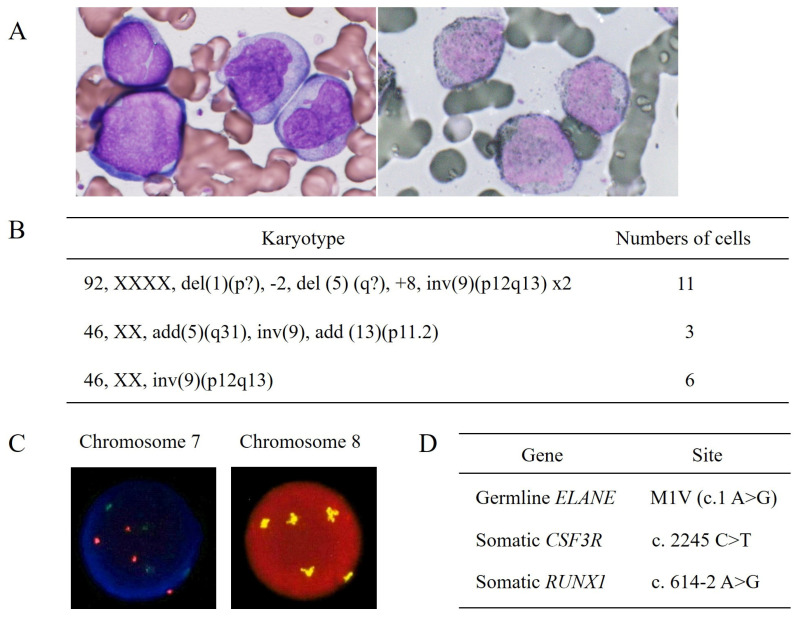
Characteristics of the leukemic blasts at the initial diagnosis of acute myeloid leukemia. (**A**) Bone marrow aspirate smears displaying blast cells by Wright–Giemsa staining (left, 1000×) and positive myeloperoxidase staining (right, 1000×). The patient was diagnosed with acute myeloid leukemia without maturation according to the WHO classification. (**B**) Karyotype of 20 cells analyzed by the Giemsa banding technique revealed abnormalities (two different types) in 70% of cells. Inversion of chromosome 9 was considered a normal variant. (**C**) Fluorescence in situ hybridization (FISH) of chromosome 7 was performed using two probes, namely D7S486 (red) recognizing the 7q31 region and D7Z1 (green) recognizing the centromere region. Excessive and equal numbers of colors were observed in 79% of the bone marrow cells, suggesting an abnormality of chromosome 7 without monosomy 7 (left). FISH of chromosome 8 was performed using D8Z2 probe, which recognizes the centromere region. Pentasomy of chromosome 8 was found in 60% of the bone marrow cells (right). The percentages of karyotypic and FISH abnormalities almost corresponded to those in the blast cells in the bone marrow aspirate smears (**A**). (**D**) DNA was extracted from the bone marrow cells and evaluated using a next-generation deep-sequencing assay. Somatic *CSF3R* and *RUNX1* mutations were identified in addition to a germline *ELANE* mutation.

The patient was referred to our hospital because of leukemic transformation with serious skin infections at 8 years of age. On admission, the patient presented with a necrotic ulcer and a protruding mass on the buttock, and numerous cutaneous scars were distributed over her entire body. Laboratory tests revealed a white blood cell (WBC) count of 1650/µL (0.5% neutrophils and 10.5% blasts), a hemoglobin level of 6.3 g/dL, a platelet count of 151,000/µL, and an elevated C-reactive protein (CRP) concentration of 3.45 mg/dL (Table 1). The serum G-CSF concentration was elevated at 162 pg/dL (normal, <39.0 pg/dL). Bone marrow examination revealed 51.5% leukemic blasts (Figure 1A, left), of which 42.5% appeared to be positive for myeloperoxidase by Giemsa counterstaining (Figure 1A, right). Flow cytometry was used to gate on blast cells that were positive for CD45 expression, which were also positive for CD13, CD15, CD33, CD34, CD117, and human leukocyte antigen (HLA)-DR, weakly positive for CD11b, and negative for cytoplasmic myeloperoxidase. Based on these characteristics, the patient was diagnosed with AML probably developed from SCN. The blasts harbored a complex karyotype (Figure 1B). Fluorescent in situ hybridization (FISH) of chromosomes 7 and 8 revealed that 79% and 60% of the bone marrow cells were found to have an abnormal chromosome 7 without monosomy 7 and pentasomy of chromosome 8, respectively (Figure 1C). The blasts harbored acquired mutations in *CSF3R* (c.2245C>T) and *RUNX1* (c.614-2A>G) (Figure 1D). Based on the characteristics of the leukemic blasts, the patient was diagnosed with AML developing from SCN.

**Figure 2:**
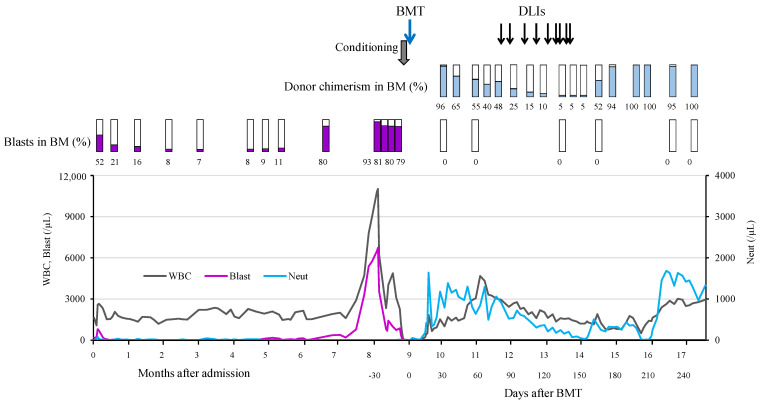
The patient’s clinical course. The clinical course is presented briefly, focusing on the period of spontaneous acute myeloid leukemia remission and BMT. Chimerism was analyzed in all bone marrow cells using DNA microsatellites at the D9S304, D16S3253, and D8S1179 loci (HLA Laboratory, Kyoto, Japan). BM: bone marrow; BMT: bone marrow transplantation; DLI: donor lymphocyte infusion; WBC: white blood cells; Neut: neutrophils.

We empirically started the patient on intravenous meropenem and micafungin with the oral administration of sulfamethoxazole/trimethoprim. Microbiological analyses were repeatedly performed; however, those cultures were not positive at all. The treatment with these antimicrobial agents improved the skin infections, accompanied by a decrease in leukemic blast counts without the administration of any chemotherapeutic agents (Figure 2). Expeditious HSCT was considered; however, no HLA-matched related or unrelated donor was identified at the time. The patient was temporarily discharged with PR and approximately 10% leukemic blasts in bone marrow. After maintaining PR for 5 months, she presented with pyrexia and severe obstructive dyspnea owing to multiple cervical lymphadenopathies. On the second admission, laboratory tests revealed a WBC count of 10,810/µL with 0% neutrophils and 60% blasts, a hemoglobin level of 7.6 g/dL, a platelet count of 57,000/µL, and an elevated CRP concentration of 12 mg/dL (Table 1). The serum G-CSF concentration was 121 pg/dL. Computed tomography revealed profound adenoid–tonsillar hypertrophy and multiple enlarged cervical lymph nodes. Bone marrow examination revealed 90% leukemic blasts with similar characteristics to those of the initial blasts. Karyotyping of 20 bone marrow cells identified abnormalities in 35% of cells with two different types that were comparable with those of the initial blasts. The rates of chromosome 7 abnormality and pentasomy 8 exceeded 85%. She underwent urgent percutaneous tracheostomy and cervical lymph node biopsy, which revealed reactive lymphoid hyperplasia without leukemic cells. Her clinical condition improved after treating the infections empirically with the intravenous administration of cefozopran, teicoplanin, and voriconazole, with no further progression of the leukemic blasts. Causative microorganisms for the infections were not identified. An HLA 7/8-matched unrelated female donor was found through the Japan Marrow Donor Program, and informed consent was obtained from the patient’s guardians to proceed with BMT. Data collection for this study was approved by the institutional review board at Hiroshima University.

To reduce leukemic blast counts, etoposide (50 mg/m^2^) was administered for 2 days starting 1 week before administering the conditioning regimen, and a decrease in the number of blasts in peripheral blood was observed. The conditioning regimen comprised total body irradiation (3.6 Gy), fludarabine (100 mg/m^2^), melphalan (180 mg/m^2^), antithymocyte globulin (5.0 mg/kg), and etoposide (520 mg/m^2^). She received 4.7 × 10^8^/kg nucleated cells and 2.8 × 10^6^/kg CD34+ cells from the donor. Tacrolimus and short-term methotrexate were used as prophylaxis against graft-versus-host disease (GVHD). Neutrophils engrafted on day 16. The rate of donor chimerism in all bone marrow cells quantitatively measured by short tandem repeat analysis exceeded 95% on day 27. However, serial analyses of chimerism revealed a gradual decrease in the rate of donor chimerism in bone marrow (Figure 2). Tacrolimus was discontinued on day 47. Donor lymphocyte infusions (DLIs) were then performed nine times from days 70 to 135. Complete donor chimerism was successfully accomplished on day 195 after nine DLIs. The patient developed grade II acute GVHD (skin, stage 3) 2 weeks after the last DLI, and the administration of prednisolone (0.8 mg/kg) was started on day 198 after confirming complete donor chimerism. The treatment gradually resolved the skin rashes, and prednisolone was discontinued on day 238. Abnormalities in chromosomes 7 and 8 were not detected in her bone marrow after transplantation. The obstructive respiratory symptoms improved with the increased rate of donor chimerism, and the tracheostomy was closed on day 225. She was discharged on day 259. Serial chimerism analyses revealed the continuation of complete donor chimerism for more than 4 years after BMT without serious infections and AML relapse.

## 3. Discussion and Conclusions

Most patients with SCN benefit from G-CSF therapy, suggesting that their prognosis was improved [12]. However, approximately 10% of patients who are mainly G-CSF non-responders still die from severe bacterial infections [13]. Furthermore, SCN is a preleukemic disease with a high risk of progression to MDS/AML, and the risk is considered to increase in association with exposure to G-CSF [7,8,13]. Among patients with SCN, HSCT is currently indicated for those with an insufficient neutrophil response to G-CSF, those requiring high-dose G-CSF therapy, those with severe infections with poor control, and those who develop MDS/AML [10,14,15].

We reported a patient with AML arising from SCN who successfully underwent BMT without standard induction chemotherapy for AML. Allogeneic HSCT is the only curative treatment for patients with SCN who develop MDS/AML [9,10,11]. Before 2000, almost all the reported patients with SCN developing AML who received standard chemotherapy for AML followed by HSCT had poor outcomes [9,16]. However, since 2001, the prognosis of these patients was improved by implementing a short interval between the diagnosis of leukemia and HSCT, with mild chemotherapy [16]. The 2015 report of the European Society for Blood and Bone Marrow Transplantation (EBMT) demonstrated that the presence of MDS/acute leukemia at transplantation was not associated with significantly reduced overall survival [14]. In this report, 16% of patients had transformed to MDS/acute leukemia before HSCT; however, information on transformation was only available for 64% of the 136 patients who underwent HSCT, and no details on the remission status of leukemia were available. Our patient received low-dose etoposide to reduce the burden and confirm the susceptibility of the leukemic blasts, followed by successful BMT using a conditioning regimen containing etoposide. Immediate HSCT following minimal chemotherapy to reduce the leukemic blast load is considered feasible for patients with SCN who develop leukemia.

Our patient did not respond to G-CSF treatment, and she experienced recurrent infections. She had an *ELANE* mutation, and when AML developed, the leukemic cells harbored both *CSF3R* and *RUNX1* mutations. Skokowa et al. reported that patients with SCN who acquire somatic *CSF3R* mutations and secondary additional mutations in *RUNX1* have a high risk of progression to MDS/AML, and these two mutations are suggested to cooperate as drivers of leukemogenesis [17]. In our patient, the finding that leukemic blasts acquired both *CSF3R* and *RUNX1* mutations in addition to the abnormal karyotypes suggested leukemic transformation in the course of SCN. The mutation in the *ELANE* gene identified in this case is reported to be refractory to G-CSF therapy [18,19]; however, the contribution of this mutation to leukemic transformation is unknown. Intriguingly, the percentage of leukemic blasts in the patient’s bone marrow decreased spontaneously from 50% to 10% with improvement in the infections after antimicrobial therapy. There are similar reports of the spontaneous remission of AML associated with infectious diseases, mainly in adults, implying the involvement of the immune system in the mechanism of remission [20,21]. Most patients experienced transient remission. These reports suggest that the treatment of infections and fever may contribute to the spontaneous remission of AML; however, no patients with AML developed from SCN were noted. Jeha et al. reported a patient with SCN who developed AML after 9 years of G-CSF treatment, and G-CSF discontinuation resulted in a spontaneous decrease in leukemic blasts without chemotherapy [22]. The acquisition of *CSF3R* mutations generates truncated G-CSF receptors, which can lead to hypersensitivity to G-CSF and/or clonal proliferation [23]. Ahmed and Lambert reported that a 5-month-old boy with SCN presented with adenoid–tonsillar hypertrophy that they speculated was related to 3 months of G-CSF administration [24]. G-CSF is considered to promote lymphocyte proliferation because both T cells and B cells express G-CSF receptors [25]. Our patient developed severe adenoid–tonsillar hypertrophy and multiple cervical lymphadenopathies at the time of AML recurrence. The role of the increased serum G-CSF level remains unclear in the course of this case; however, it is likely that the increased G-CSF level may have played a role in the progression of leukemia and lymphoid hyperplasia. Thus, it appears that the clinical manifestations and course of leukemic progression in patients with SCN may be highly variable. The control of neutropenia-related infections is necessary to carefully observe leukemia progression and improve patients’ general condition prior to HSCT. Moreover, achieving complete donor chimerism is important for avoiding the reappearance of clones predisposed to leukemia derived from recipients in bone marrow [14].

The EBMT study also reported that the 3-year overall survival rate following HSCT was significantly higher in SCN patients below 10 years of age compared with those older than 10 years (87% vs. 68%; *p* = 0.016), which may be explained by their lower exposure to infections [14]. Although HSCT for our patient was first proposed years before it eventually took place, her parents declined it because she had not yet experienced life-threatening infections. On her first admission to our hospital, HSCT was considered unavoidable; however, no appropriate donor was found at the time. She finally underwent HSCT at 9 years of age.

The conditioning regimen used in this case induced relatively early neutrophil engraftment while resulting in mixed chimerism. Nine DLIs led to the achievement and maintenance of complete donor chimerism without leukemia relapse for more than 4 years. HSCT with preferably myeloablative conditioning for maintaining complete donor chimerism following minimal chemotherapy is considered important for curing patients with AML arising from SCN.

In summary, a patient with SCN carrying an *ELANE* mutation developed AML that acquired mutations in *CSF3R* and *RUNX1*. The leukemic blast counts initially decreased concomitantly with treatment of the infections. After AML recurrence, the patient was successfully treated with HSCT using modified chemotherapy and a conditioning regimen. When MDS/AML develops in patients with SCN, it is important initially to stabilize the patients’ general condition and then proceed to HSCT following minimal chemotherapy with conditioning that leads to complete donor chimerism.

## Figures and Tables

**Table 1:** Laboratory findings at different timepoints.

	First Admission	First Discharge	Second Admission	Days after Transplantation				Normal Range
				257	600	1290	1654	
WBCs (/µL)	1650	1510	10,810	2950	3230	5230	4920	3300–8600
Neutrophils (%)	0.5	0	0	45	53	58	60	38.5–81.5
Blasts (%)	10.5	4	60	0	0	0	0	
Hb (g/dL)	6.3	7.0	7.6	9.2	12.4	12.8	12.3	11.6–14.8
Platelets (/µL)	151,000	412,000	57,000	208,000	201,000	204,000	219,000	158,000–348,000
CRP (mg/dL)	3.45	1.73	12.0	0.05	0.07	0.02	0.01	0–0.14
G-CSF (pg/dL)	162		121					<39.0

WBCs: white blood cells; Hb: hemoglobin; CRP: C-reactive protein; G-CSF: granulocyte colony-stimulating factor.

## Data Availability

The data that support the findings of this study are available from the corresponding author upon reasonable request.
